# PET/CT-Based prognostic model enhances early survival prediction in angioimmunoblastic t-cell lymphoma

**DOI:** 10.3389/fimmu.2025.1607177

**Published:** 2025-07-16

**Authors:** Xiaoxia Xu, Xiangxiang Ding, Xiao Jin, Yunfei Shi, Zhi Yang, Nan Li

**Affiliations:** ^1^ Key laboratory of Carcinogenesis and Translational Research (Ministry of Education), Beijing Key Laboratory of Research, Investigation and Evaluation of Radiopharmaceuticals, NMPA Key Laboratory for Research and Evaluation of Radiopharmaceuticals (National Medical Products Administration), Department of Nuclear Medicine, Peking University Cancer Hospital & Institute, Beijing, China; ^2^ Key Laboratory of Carcinogenesis and Translational Research (Ministry of Education/Beijing), Department of Pathology, Peking University Cancer Hospital & Institute, Beijing, China

**Keywords:** angioimmunoblastic T-cell lymphoma, PET/CT, overall survival, prediction model, cox regression model

## Abstract

**Background:**

To develop and validate a new prognostic model using baseline PET parameters and clinical indicators for predicting early overall survival (OS) in Angioimmunoblastic T-cell lymphoma (AITL) patients.

**Methods:**

We conducted a retrospective cohort study from December 2009 to December 2023 (n=124) at a single center. The model’s predictors included baseline clinical characteristics, pathological indicators, laboratory metrics, and PET/CT parameters. Independent prognostic factors were identified using Cox regression and presented as nomograms. The C-index assessed predictive accuracy, while calibration plots and decision curve analysis evaluated prediction accuracy and discrimination ability. The model’s accuracy was compared with existing prognostic systems using C-index, NRI, ROC, and Kaplan-Meier survival curves.

**Results:**

SUVmax, β2MG, platelet, and albumin were identified as independent risk factors. The C-index for OS was 0.78 (95% CI: 0.70-0.85); for 1000 bootstrap samples, it was 0.76 (95% CI: 0.61-0.93). Calibration curves showed excellent agreement between predictions and actual observations. The AUC for 6-month and 1-year OS were 0.91(95% CI: 0.82-1.00) and 0.85 (95% CI: 0.77–0.94), respectively. The model outperformed PIAI, IPI, and PIT in predictive capacity.

**Conclusion:**

The new prediction model reliably estimates outcomes for AITL patients, demonstrating high discrimination and calibration.

## Introduction

Angioimmunoblastic T-cell lymphoma (AITL) is an uncommon hematologic cancer arising from mature T follicular helper cells. It ranks as the second most prevalent subtype of peripheral T-cell lymphoma, comprising about 1%–2% of non-Hodgkin’s lymphoma (1, 2). AITL is clinically characterized by an aggressive course. Although chemotherapy, targeted therapy, immunotherapy, and other therapeutic methods have been applied in the treatment of AITL, this malignancy tends to progress rapidly and carries a poor prognosis, with a 5-year OS rate ranging between 32–33% (3–5). Therefore, the early identification of high-risk patients is crucial for better management and improved outcomes.

Current risk assessment tools, including the International Prognostic Index (IPI) (6) and the Prognostic Index for T-cell lymphoma (PIT) (7) are suboptimal for AITL as they were developed for broader lymphoma subtypes (8). However, these tools demonstrate limited accuracy in AITL due to biological heterogeneity (4). While the AITL-specific PIAI score models (4, 5) represent advances, they rely exclusively on clinical parameters (e.g., age, LDH, bone marrow involvement) and lack functional imaging biomarkers. This limits their ability to capture tumor microenvironment heterogeneity and metabolic tumor burden—key factors in AITL pathogenesis (9).

In recent years, the utility of FDG PET/CT in lymphoma management has been well-established (10–12). Numerous imaging-derived quantitative parameters have been reported for their potential use in predicting prognosis and treatment outcomes in Hodgkin’s lymphoma and diffuse large B-cell lymphoma (12–14). These metrics include those based on tumor volume and metabolic features. However, its role in AITL remains underexplored: only few small studies (15, 16) have evaluated baseline PET/CT parameters, and none have integrated them into multivariate prognostic models.

To bridge this gap, we developed and validated a novel PET/CT-enhanced prognostic model for early survival prediction in AITL. Using a retrospective single-center cohort, we (1): identified independent clinical and PET/CT predictors via Cox regression (2); constructed a nomogram for 1-year OS prediction (3); internally validated the model’s accuracy and clinical utility. This tool aims to facilitate risk-adapted therapeutic strategies and clinical trial design for this high-risk lymphoma subtype.

## Methods

### Patients

Between December 2009 and December 2023, consecutive patients newly diagnosed with AITL according to WHO classification criteria were recruited for this retrospective study. Patients without a baseline PET/CT scan were excluded. Patients with concomitant infections or other malignant tumors were also excluded from the study. All research procedures were in accordance with the requirements of the hospital’s ethics committee. Data were collected from hospital medical records. Written informed consent was waived based on all of the following criteria: 1) Retrospective design: Analysis of pre-existing clinical data collected during standard care. 2)Minimal risk: No additional interventions; data anonymization ensured no personal identifiers were disclosed. 3) Impracticability: Infeasibility of re-contacting patients treated over 14-year span (2009–2023).

### PET/CT scan and image analysis

All included patients underwent whole-body ^18^F-FDG PET/CT scans using a PET/CT system. After fasting for at least 6 hours and ensuring blood glucose levels were below 200 mg/dL, an intravenous injection of 3.7 MBq/kg of ^18^F-FDG was administered. Approximately 60 minutes after the ^18^F-FDG injection, whole-body CT and PET scans were acquired. The acquired CT and PET images were reviewed on a dedicated Siemens workstation (Syngo.via VB20, MM Oncology). PET/CT images were retrospectively reviewed by two experienced nuclear medicine physicians. An iso-activity contour was automatically drawn in the VOI using the preset margin threshold, and SUVmax, metabolic tumor volume (MTV) and total lesion glycolysis (TLG) were calculated. MTV was obtained with a fixed 41% SUVmax threshold according to the EANM recommendations (17, 18), the isocontour described as 41% generally corresponds best with the actual dimensions of the tumor, although only for higher tumour-to-background values and homogenous backgrounds (17).

### Variable collection

The present study included several variables: baseline clinical characteristics (age at diagnosis, Ann Arbor stage, performance status, extranodal sites, bone marrow involvement, B symptom), baseline pathological indicators (EBV-encoded RNA, Ki67), baseline laboratory indicators (lactate dehydrogenase, beta 2-microglobulin (β2MG), platelet, albumin, baseline PET/CT parameters (SUVmax, MTV, TLG), and survival variables (vital status, survival months). The primary outcome in our research was OS, defined from the date of diagnosis to death or the last follow-up. At presentation, the IPI, PIT and PIAI were collected for comparation. The standard IPI includes age ≥ 60 years, stages III to IV disease, lactate dehydrogenase > normal, extranodal sites > one, and performance status ≥ 2. The PIT includes age ≥ 60 years, performance status ≥ 2, lactate dehydrogenase > normal, and bone marrow involvement. The PIAI includes age > 60 years, performance status ≥ 2, extranodal sites > one, B symptoms, and platelet count < 150 × 10^9/L (4, 19, 20).

### Statistical analysis

The Cox proportional hazard regression model estimated the hazard ratio (HR) and corresponding 95% confidence interval (CI) for each potential risk factor. Concurrently, 1000 bootstrap samples were used for model validation. Variables with *P*<0.05 in univariate Cox regression were included in multivariate analysis. The backward method was applied in the multivariate model to construct the risk prediction model. Meanwhile, we randomly divided the cohort (n=124) into a training set of 87 patients (70%) and a validation set of 37 patients (30%), and implemented strict internal validation.

The developed models were validated for their discriminative ability using C-statistics. Selected variables were incorporated into nomograms to predict early (6-month, 1-year) OS of patients with AITL. Survival curves were analyzed using the Kaplan-Meier method and compared with the log-rank test according to prognostic factors. The optimal cut-off values of selected quantitative parameters for survival prediction were determined using the receiver operating characteristic (ROC) curve and the best Youden index.

The Kaplan-Meier survival curve, C-index, Net reclassification index (NRI), the ROC curve and Time-dependent ROC curve were used to evaluate the discrimination of the nomogram and to contrast the predictive and discriminatory capacity of the nomograms against with PIAI, IPI and PIT. Calibration was evaluated by a calibration plot, which compares the relationship between the predicted and observed probabilities for survival time. The clinical usefulness and benefits of the nomogram were estimated by the decision curve analysis. Statistical analysis was performed using R version 4.4.1 (http://www.R-project.org) along with Storm Statistical Platform (www.medsta.cn/software). A *P* value less than 0.05 was considered statistically.

## Results

### Patient characteristics

The baseline clinical characteristics of the 124 patients enrolled in this study were listed in **Table 1**. Among these patients, 2 (1.61%) had Ann Arbor stage I, 8 (6.45%) had Ann Arbor stage II, 56 (45.16%) had Ann Arbor stage III, and 58 (46.77%) had Ann Arbor stage IV. Additionally, 55 (44.35%) exhibited extranodal involvement, and 21 (16.94%) had bone marrow involvement at baseline diagnosis. The majority of patients were treated with cyclophosphamide, doxorubicin, vincristine, and prednisone-based protocol as first line therapy. The median OS was 15 months, with a median follow-up of 36 months.

**Table 1:** The baseline clinical characteristics.

Patients Characteristics	Total (n = 124)
Age, n(%)	
<=60y	47 (37.90)
>60y	77 (62.10)
Gender, n(%)	
Male	82 (66.13)
Female	42 (33.87)
Extranodal sites, n(%)	
No	69 (55.65)
Yes	55 (44.35)
Bone marrow involvement, n(%)	
No	103 (83.06)
Yes	21 (16.94)
Performance status, n(%)	
<2	105 (84.68)
>=2	19 (15.32)
Stage, n(%)	
I	2 (1.61)
II	8(6.45)
III	56(45.16)
IV	58(46.77)
PIAI, n(%)	
0	13 (11.21)
1	41 (35.34)
2	27 (23.28)
3	20 (17.24)
4	13 (11.21)
5	2 (1.72)
PIT, n(%)	
0	13 (11.71)
1	46 (41.44)
2	31 (27.93)
3	18 (16.22)
4	3 (2.70)
IPI, n(%)	
0	2 (1.79)
1	14 (12.50)
2	29 (25.89)
3	38 (33.93)
4	23 (20.54)
5	6 (5.36)

### Prognostic factors of OS

A total of 15 parameters were included in the set. Using OS state as a grouping variable, the basic statistical analysis of the total set was presented in **Table 2**. SUVmax, β2MG, and lactate dehydrogenase showed significant differences with *P* values of <0.001, 0.011, and 0.016, respectively. According to the univariate and multivariate analysis for OS, variables considered prognostic factors were summarized in **Table 3**. In the univariate analysis, high β2MG, low platelet, low albumin, and low SUVmax were significant factors for unfavorable OS, with *P* values of 0.003 (HR:2.83, 95%CI:1.41-5.67), 0.003 (HR: 2.61, 95%CI: 1.39-4.89), 0.007(HR:2.72, 95%CI:1.32-5.59), and 0.037(HR:0.95, 95%CI:0.90-0.99), respectively. The Akaike Information Criterion using the Cox proportional hazard model was applied to determine the strongest predictors. High β2MG, low platelet, low albumin, and low SUVmax were also significantly associated with unfavorable OS (**Table 3**). The *P* values for these factors were 0.011 (HR: 2.60, 95%CI: 1.25-5.43), 0.003 (HR: 3.41, 95%CI:1.50-7.72), 0.023 (HR: 2.58, 95%CI:1.14-5.84), and <0.001 (HR: 0.88, 95%CI:0.83-0.94), respectively.

**Table 2:** Basic characteristics and difference analysis of total set.

Variables	Total (n = 124)	0 (n = 75)	1 (n = 49)	Statistic	*P*
Age, n(%)				χ²=1.83	0.176
<=60y	47 (37.90)	32 (42.67)	15 (30.61)		
> 60y	77 (62.10)	43 (57.33)	34 (69.39)		
Performance status, n(%)				χ²=0.07	0.796
<2	105 (84.68)	63 (84.00)	42 (85.71)		
>=2	19 (15.32)	12 (16.00)	7 (14.29)		
Stage, n(%)				χ²=2.74	0.098
I-II	10 (8.06)	9 (12.00)	1 (2.04)		
III-IV	114 (91.94)	66 (88.00)	48 (97.96)		
Extranodal sites, n(%)				χ²=0.22	0.640
No	69 (55.65)	43 (57.33)	26 (53.06)		
YES	55 (44.35)	32 (42.67)	23 (46.94)		
Marrow involvement, n(%)				χ²=0.12	0.731
No	103 (83.06)	63 (84.00)	40 (81.63)		
Yes	21 (16.94)	12 (16.00)	9 (18.37)		
B symptom, n(%)				χ²=0.05	0.825
No	68 (56.20)	41 (55.41)	27 (57.45)		
Yes	53 (43.80)	33 (44.59)	20 (42.55)		
Ki67, n(%)				χ²=0.92	0.338
Low (<0.33)	31 (26.05)	21 (29.17)	10 (21.28)		
High (>=0.33)	88 (73.95)	51 (70.83)	37 (78.72)		
EBV-encoded RNA, n(%)				χ²=0.61	0.435
Negative (-)	30 (26.79)	20 (29.41)	10 (22.73)		
Positive (+)	82 (73.21)	48 (70.59)	34 (77.27)		
β2MG, n(%)				χ²=6.53	0.011
Low (<3.23mg/L)	47 (44.76)	35 (54.69)	12 (29.27)		
High (>=3.23mg/L)	58 (55.24)	29 (45.31)	29 (70.73)		
Lactate dehydrogenase, n(%)				χ²=5.78	0.016
Low (<312.5IU/L)	72 (64.86)	50 (73.53)	22 (51.16)		
High (>=312.5IU/L)	39 (35.14)	18 (26.47)	21 (48.84)		
Platelet, n(%)				χ²=1.56	0.211
Low (<150*10^9/L)	23 (19.49)	11 (15.71)	12 (25.00)		
High (>=150*10^9/L)	95 (80.51)	59 (84.29)	36 (75.00)		
Albumin, n(%)				χ²=1.30	0.254
Low (<35.0g/L)	20 (18.02)	10 (14.71)	10 (23.26)		
High (>=35.0g/L)	91 (81.98)	58 (85.29)	33 (76.74)		
SUVmax, Mean ± SD	12.98 ± 7.62	14.71 ± 8.62	10.32 ± 4.71	t=3.66	<.001
MTV, Mean ± SD	295.03 ± 274.79	286.46 ± 266.20	308.15 ± 289.76	t=-0.43	0.669
TLG, Mean ± SD	1484.26 ± 2284.94	1707.13 ± 2742.67	1143.12 ± 1259.17	t=1.35	0.180

t, t-test; χ², Chi-square test; SD, standard deviation. Bold values in the “P” column indicate statistically significant results (P < 0.05).

**Table 3:** Results of univariate and multivariate cox proportional risk models.

Variables	univariate					multivariate				
	β	S.E	Z	*P*	HR (95%CI)	β	S.E	Z	*P*	HR (95%CI)
Age, n(%)										
<=60y					1.00 (Reference)					
> 60y	0.54	0.31	1.73	0.083	1.71 (0.93 ~ 3.15)					
Performance status, n(%)										
<2					1.00 (Reference)					
>=2	0.45	0.41	1.09	0.275	1.57 (0.70 ~ 3.55)					
Stage										
I-II					1.00 (Reference)					
III-IV	1.42	1.01	1.41	0.159	4.15 (0.57 ~ 30.10)					
Extranodal sites, n(%)										
No					1.00 (Reference)					
Yes	0.36	0.29	1.23	0.218	1.43 (0.81 ~ 2.52)					
Marrow involvement										
No					1.00 (Reference)					
Yes	0.11	0.37	0.30	0.763	1.12 (0.54 ~ 2.31)					
B symptom, n(%)										
No					1.00 (Reference)					
Yes	-0.09	0.30	-0.31	0.760	0.91 (0.51 ~ 1.64)					
Ki67, n(%)										
Low (<0.33)					1.00 (Reference)					
High (>=0.33)	0.21	0.36	0.60	0.550	1.24 (0.61 ~ 2.50)					
EBV-encoded RNA										
Negative (-)					1.00 (Reference)					
Positive (+)	0.59	0.36	1.64	0.101	1.81 (0.89 ~ 3.69)					
β2MG, n(%)										
Low (<3.23mg/L)					1.00 (Reference)					1.00 (Reference)
High (>=3.23mg/L)	1.04	0.36	2.93	**0.003**	2.83 (1.41 ~ 5.67)	0.96	0.38	2.55	**0.011**	2.60 (1.25 ~ 5.43)
Lactate dehydrogenase, n(%)										
Low (<312.5IU/L)					1.00 (Reference)					
High (>=312.5IU/L)	0.44	0.31	1.45	0.148	1.56 (0.85 ~ 2.83)					
Platelet, n(%)										
Low (<150*10^9/L)	0.96	0.32	2.99	**0.003**	2.61 (1.39 ~ 4.89)	1.23	0.42	2.94	**0.003**	3.41 (1.50 ~ 7.72)
High (>=150*10^9/L)					1.00 (Reference)					1.00 (Reference)
Albumin, n(%)										
Low (<35.0g/L)	1.00	0.37	2.72	**0.007**	2.72 (1.32 ~ 5.59)	0.95	0.42	2.27	**0.023**	2.58 (1.14 ~ 5.84)
High (>=35.0g/L)					1.00 (Reference)					1.00 (Reference)
SUVmax	-0.05	0.02	-2.09	0.037	0.95 (0.90 ~ 0.99)	-0.12	0.03	-3.80	<.001	0.88 (0.83 ~ 0.94)
MTV	0.00	0.00	0.23	0.822	1.00 (1.00 ~ 1.00)					
TLG	-0.00	0.00	-1.27	0.204	1.00 (1.00 ~ 1.00)					

HR, Hazards Ratio; CI, Confidence Interval. Bold values in the "P" column indicate statistically significant results (P < 0.05).

**Figure 1:**
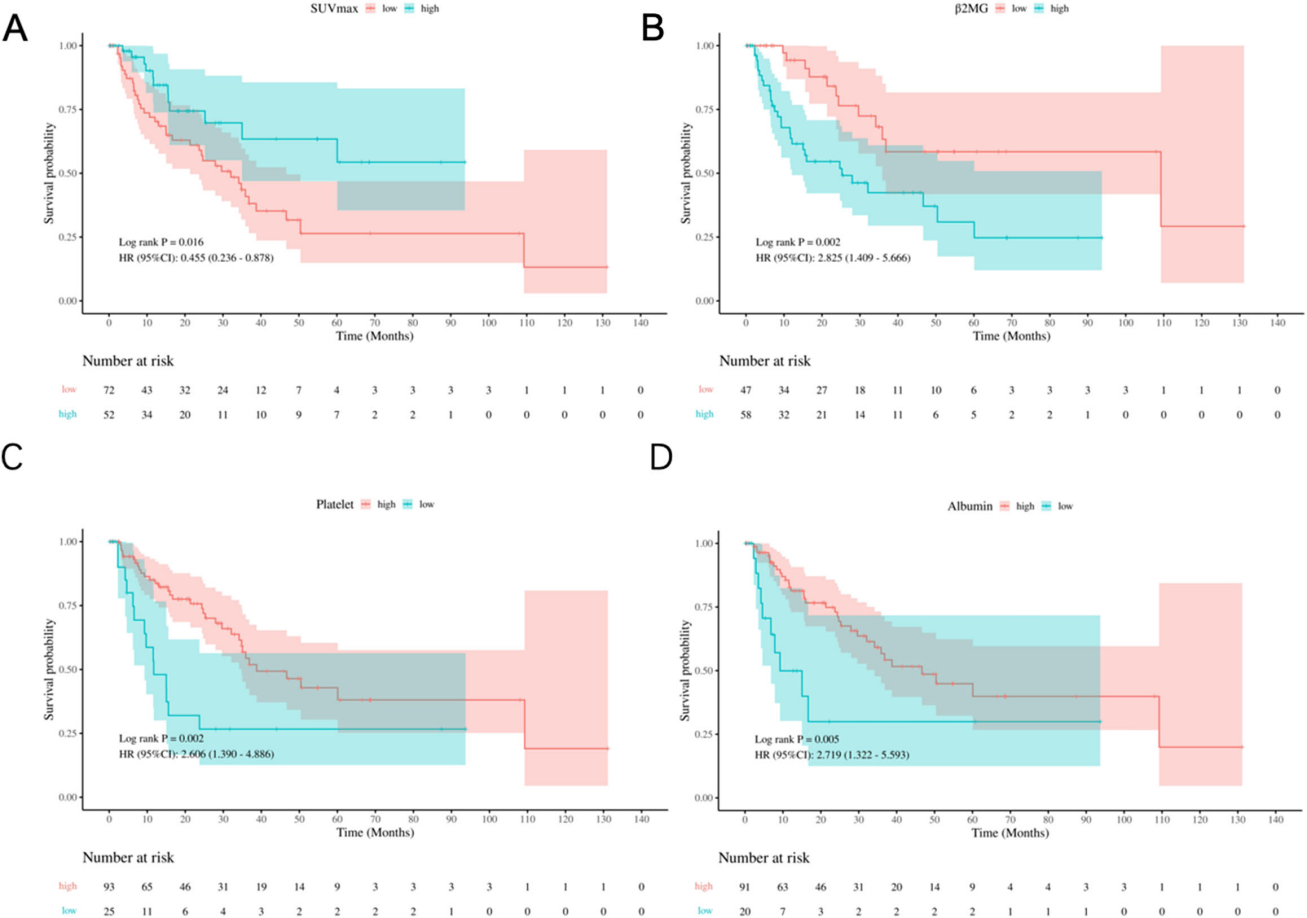
Kaplan-Meier survival analysis of prognostic variables. **(A)** OS by SUVmax groups (low: <12.95, high: ≥12.95). **(B)** OS by β2MG groups (low: <3.23 mg/L; high: ≥3.23 mg/L). **(C)** OS by platelet count groups (low: <150×10^9^/L; high: ≥150×10^9^/L). **(D)** OS by albumin groups (low: <35.0 g/L; high: ≥35.0 g/L).

The study analyzed several prognostic biomarkers to assess OS using clearly defined cut-off values. The mean SUVmax was 12.23 ± 6.99. The ROC curve analysis identified 12.95 as the optimal cut-off, allowing classification into high- (≥ 12.95) and low- (< 12.95) SUVmax groups. Kaplan-Meier survival curves showed significantly lower OS rates in the low SUVmax group (Log-rank P = 0.016, HR: 0.455, 95% CI: 0.236-0.878, **Figure 1A**). Similarly, the mean β2MG level was 4.23 ± 2.87 mg/L, with a cut-off determined at 3.23 mg/L. Patients were grouped into high- (> 3.23 mg/L) and low- (≤ 3.23 mg/L) β2MG categories. Kaplan-Meier curves indicated that high β2MG was associated with significantly worse OS (Log-rank P = 0.002, HR: 2.825, 95% CI: 1.409-5.666, **Figure 1B**). For platelet levels, the mean was 195.94 ± 73.10 × 10^9/L, and the cut-off was set at 150 × 10^9/L, in line with PIAI criteria. Patients with low platelet levels (< 150 × 10^9/L) exhibited significantly inferior OS, as depicted by Kaplan-Meier survival curves (Log-rank P = 0.009, HR: 2.356, 95% CI: 1.220-4.551, **Figure 1C**). Finally, the mean albumin concentration was 40.7 ± 5.3 g/L, with the threshold set at 35.0 g/L according to clinical standards. Patients with albumin levels below 35.0 g/L had notably poorer OS (Log-rank P = 0.005, HR: 2.719, 95% CI: 1.322-5.593, **Figure 1D**), as reflected in the Kaplan-Meier survival curves. These findings highlight the prognostic value of SUVmax, β2MG, platelet, and albumin in predicting overall survival. The resulting AUC curves at early clinical timepoints (e.g., 6 months, 1 year, 2 years) demonstrate sustained and robust discriminatory ability throughout the follow-up period (**Supplementary Figure S2**).

### Nomogram construction and bootstrap validation

**Figure 2:**
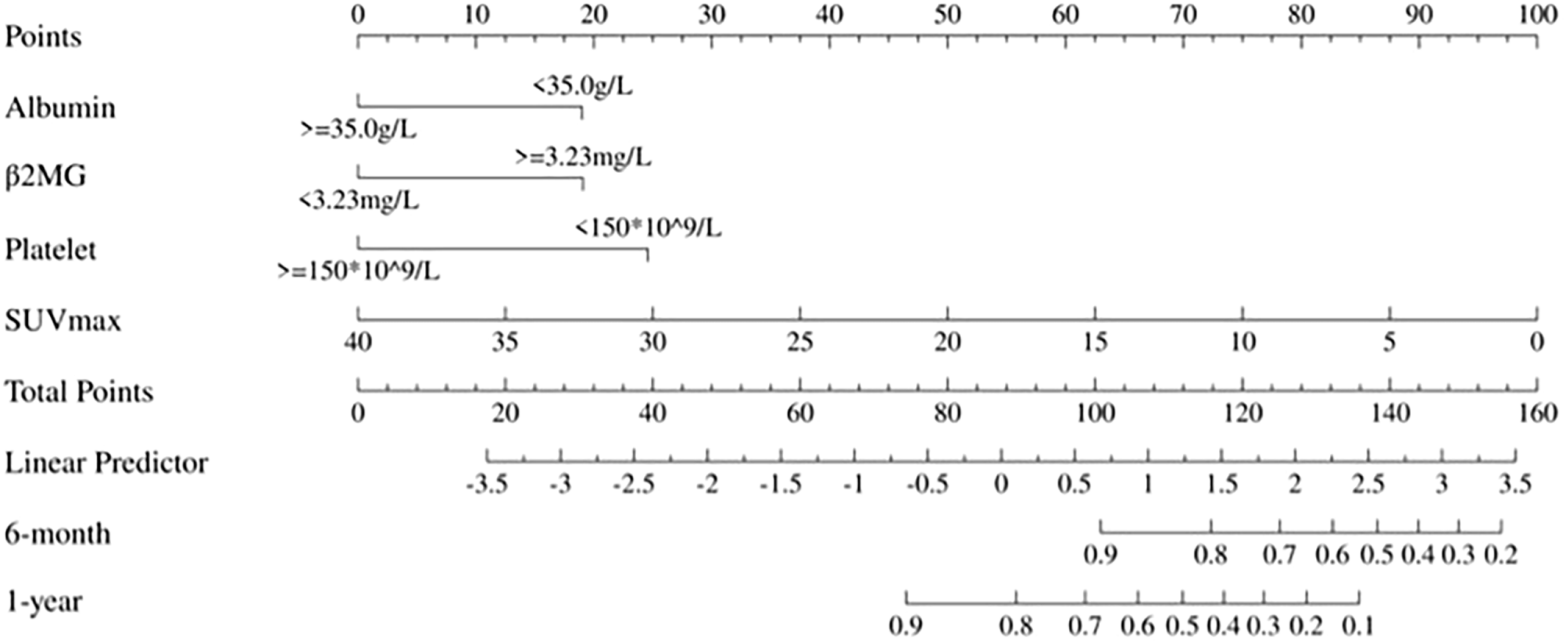
Static nomogram for 6-month and 1-year OS prediction. Point-based scoring system integrating SUVmax, β2MG, albumin, and platelet count. Total points correspond to survival probabilities.

We constructed nomograms using selected variables—β2MG, platelet, albumin, and SUVmax—to predict early OS rates at 6-month and 1-year OS for patients with AITL (**Figure 2**). The nomograms demonstrated a C-index of 0.78 (95% CI: 0.70-0.85) for OS prediction. Validation using 1000 bootstrap samples yielded a C-index of 0.76 (95% CI: 0.61-0.93). Notably, excluding PET parameters reduced the model’s C-index to 0.70 (95% CI: 0.62-0.77) based on Cox proportional hazard regression model (**Supplementary Table S1**). Additionally, the core predictors (SUVmax, β2MG, Platelet, Albumin) remained identical across split-validation approaches (Training set: 0.77 (95% CI: 0.71-0.83); Validation set: C-index = 0.84 (95% CI: 0.76-0.92)), reinforcing their biological/clinical relevance in AITL prognosis (**Supplementary Table S2**, **Supplementary Figure S1**).

**Figure 3:**
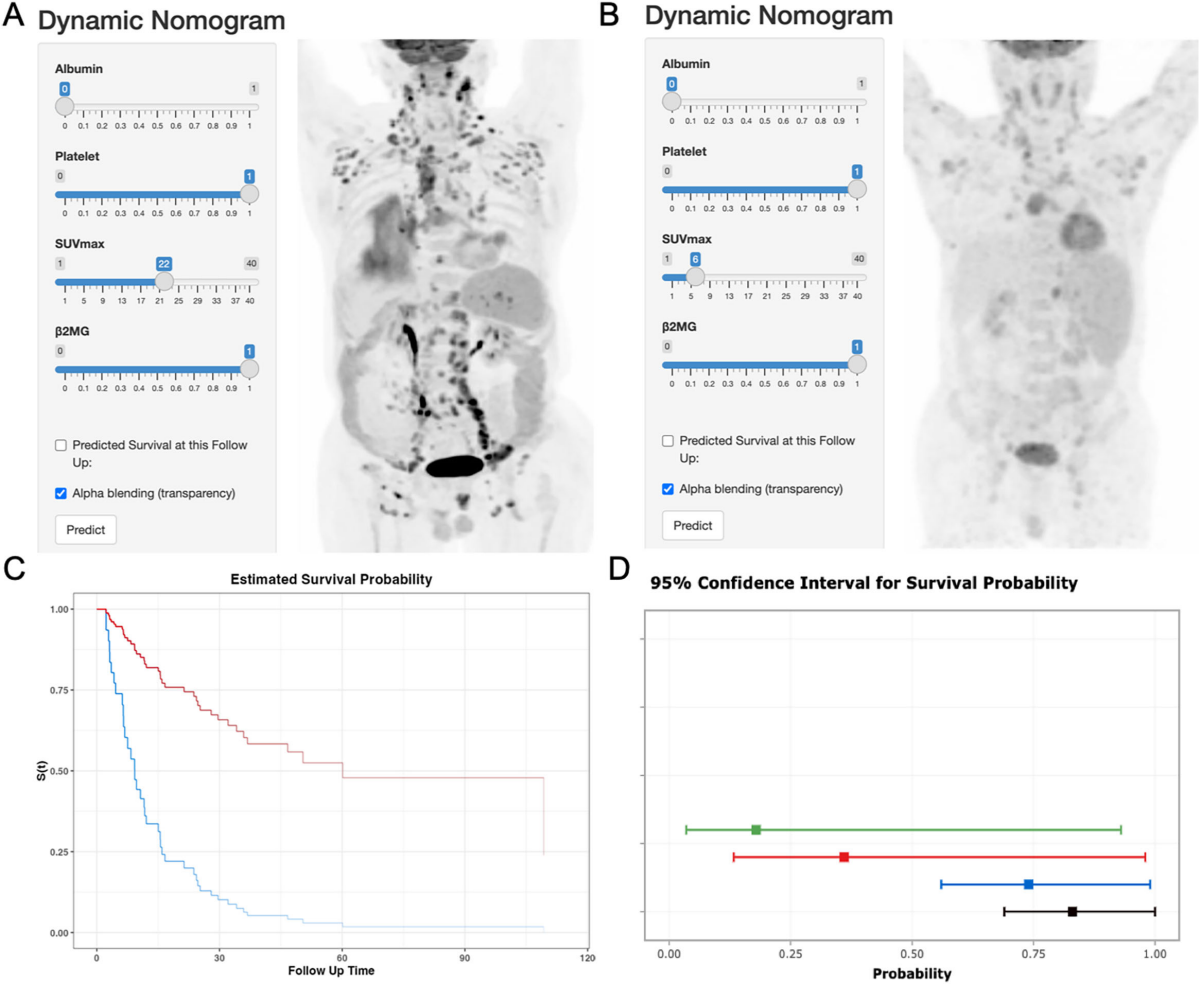
Dynamic nomogram interface and validation cases. **(A)** Interface screenshot with MIP image of Patient No.79 (66y, SUVmax=6.16, albumin=normal, platelets=low, β2MG=high). **(B)** Interface screenshot with MIP image of Patient No. 31 (70y, SUVmax=22.19, albumin=normal, platelets=low, β2MG=high). **(C)** Predicted OS curves: Patient 79 (blue), Patient 31 (red). **(D)** 95% confidence bands for survival predictions.

Each variable level was assigned a specific point in the nomogram, allowing the calculation of total points by summing across variables. These total points were then used to project early OS probabilities on the nomogram’s total score scale, leading to a dynamic nomogram for visualization. For illustration, two patients were analyzed: Patient No.79, aged 66, with SUVmax 6.16, normal albumin, low platelet, and high β2MG, had a total score of 120, corresponding to 6-month and 1-year OS rates of approximately 74.0% and 36.0%, respectively. Conversely, Patient No.31, aged 70, with SUVmax 22.19, normal albumin, low platelet, and high β2MG, scored 91, corresponding to 6-month and 1-year OS rates of about 95.0% and 83.0%, respectively. **Figure 3** illustrates the dynamic nomogram’s survival probability estimates and 95% confidence intervals (https://github.com/xxxbjmu/data.git).

**Figure 4:**
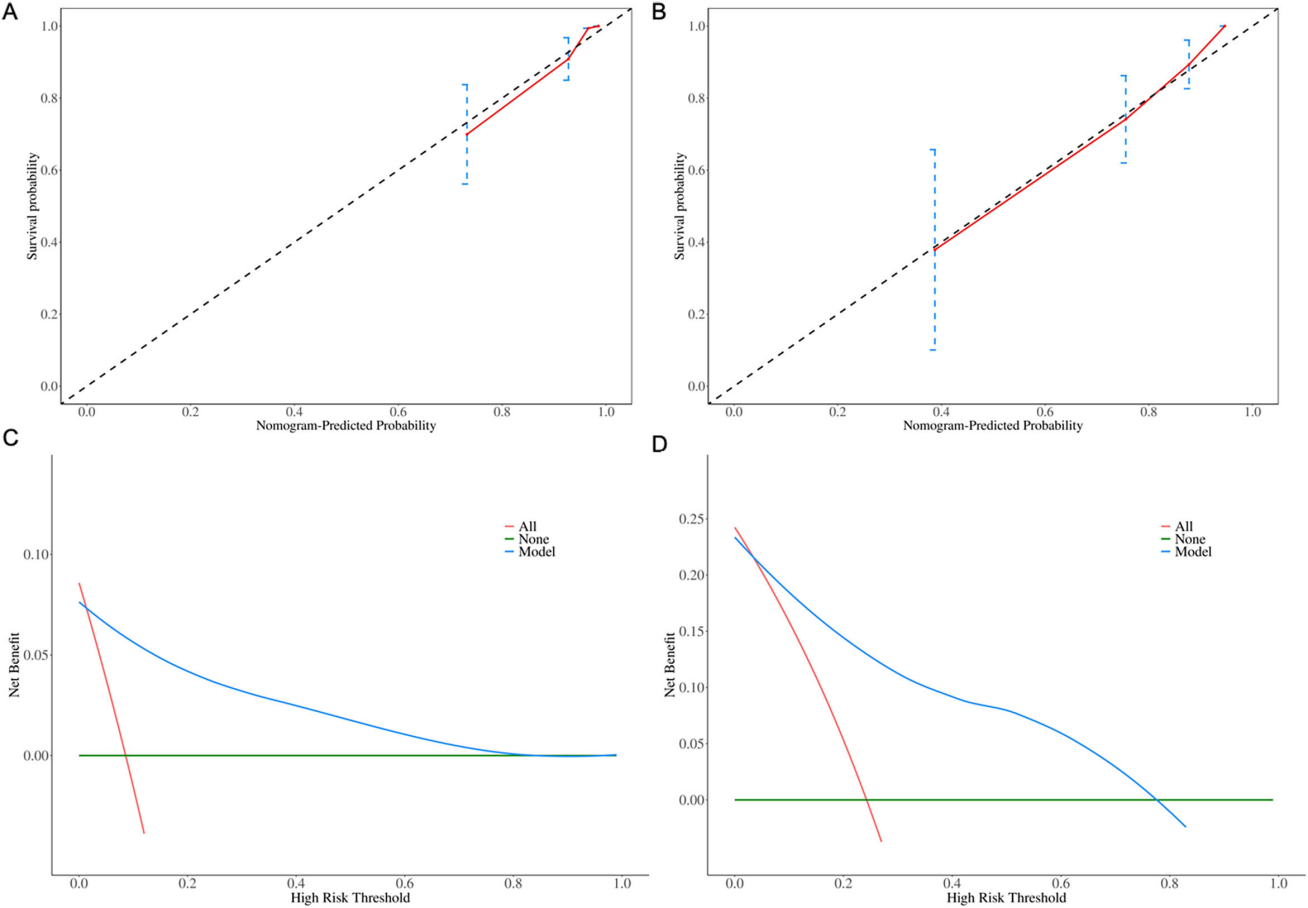
Calibration plots for **(A)** 6-month and **(B)** 1-year OS predictions. Decision curve analysis for **(C)** 6-month and **(D)** 1-year clinical utility.

The calibration plots indicated strong consistency between predicted and observed early survival probabilities (**Figures 4A, B**). To further evaluate the discriminative capacity and clinical use of the models, the decision curve analysis plots were performed (**Figures 4C, D**). The plots illustrated that the threshold probabilities of the prediction model in the sets were 2–82% (6-month) and 5–78% (1- year), indicating that the model had clinical decision-making value evaluated by balancing discrimination and calibration.

### Comparison of the predictive accuracy for OS between the nomograms and current prognostic scoring systems

**Figure 5:**
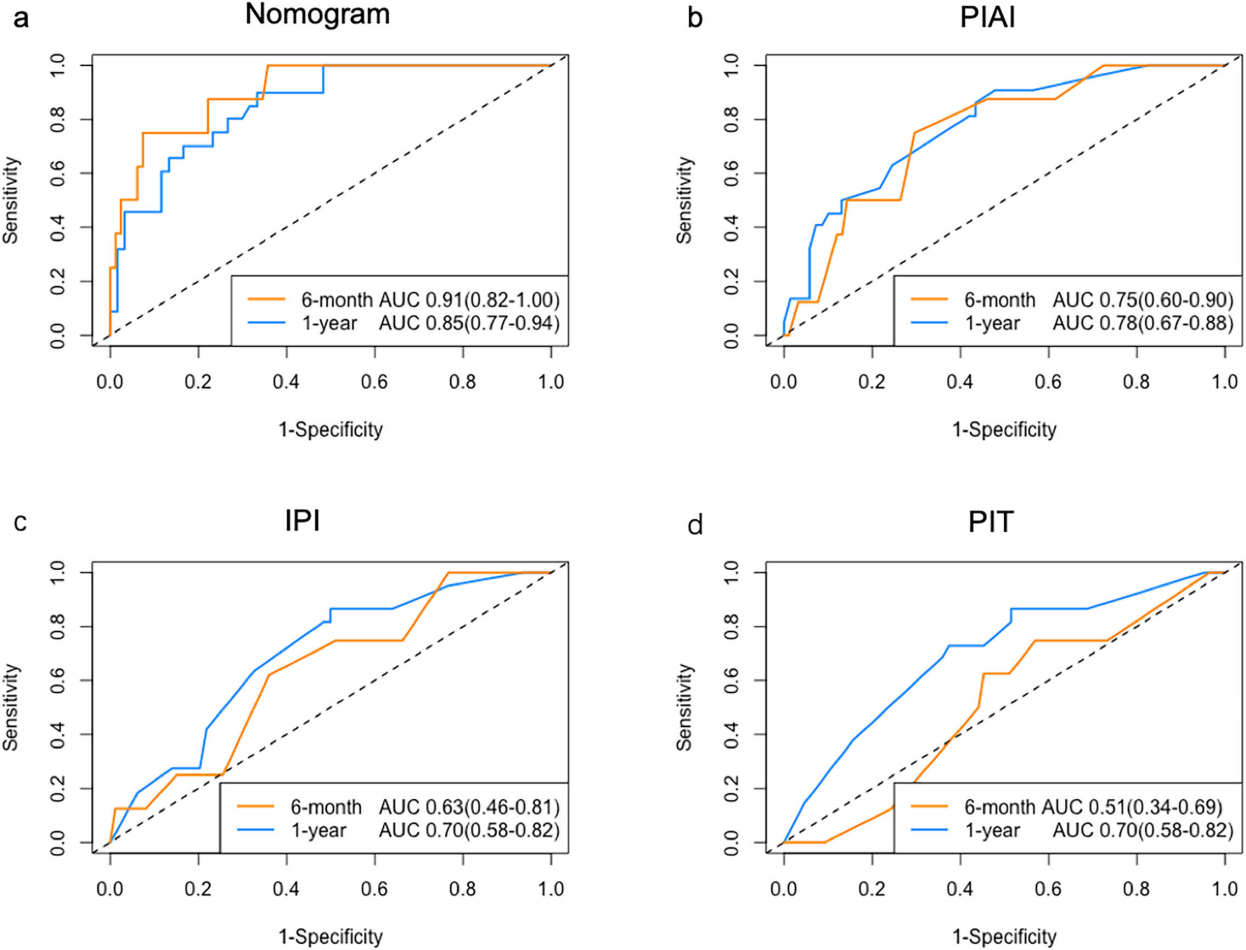
ROC analysis of prognostic models. AUC comparison for **(a)** Nomogram, **(b)** PIAI, **(c)** IPI, and **(d)** PIT in predicting early OS.

The C-index of the nomogram for OS was 0.78 (95% CI: 0.70-0.85), surpassing the PIAI, IPI and PIT, which were 0.67 (95% CI: 0.60-0.75), 0.64 (95% CI: 0.56–0.72) and 0.64 (95% CI: 0.56–0.72), respectively. Our results similarly indicated that the area under the curves (AUCs) of our prognostic nomograms were higher than those of the PIAI, IPI and PIT, particularly at early time points (**Figure 5**). Specifically, the nomogram’s AUCs at 6-month and 1-year were 0.91(95% CI:0.82–1.00) and 0.85 (95% CI: 0.77–0.94), respectively. In contrast, the AUCs of the PIAI at 6-month and 1-year were 0.75(95% CI:0.60-0.90) and 0.78 (95% CI: 0.67–0.88), respectively. For the IPI, the AUCs were 0.63(95% CI:0.46–0.81) at 6-month and 0.70 (95% CI: 0.58–0.82) at 1-year. Finally, the PIT’s AUCs were 0.51(95% CI:0.34–0.69) at 6-month and 0.70 (95% CI: 0.58–0.82) at 1-year. These findings suggest that our nomograms could be more accurate and useful tools for the prognosis of early OS in patients with AITL.

**Figure 6:**
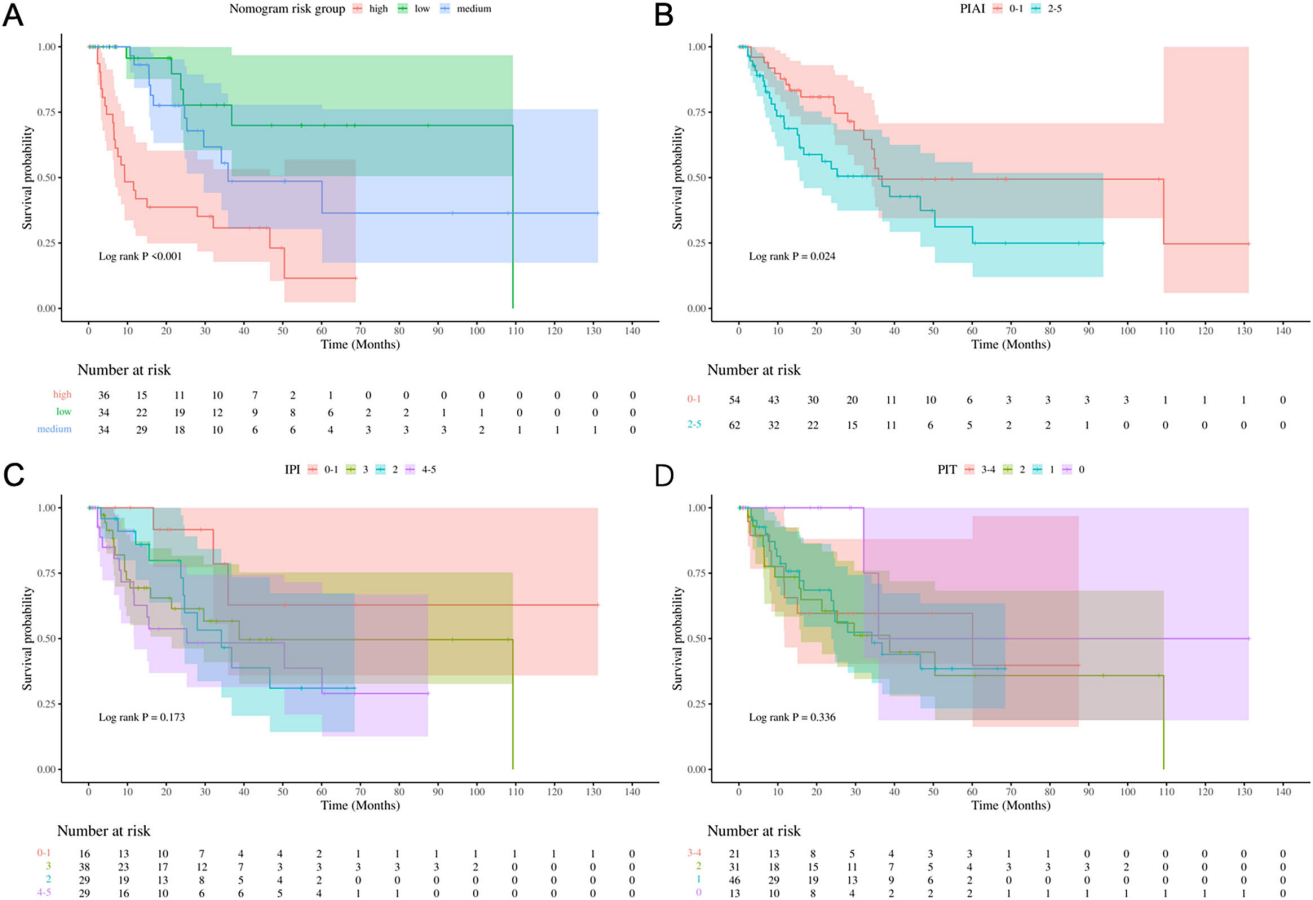
Risk stratification performance. Kaplan-Meier curves stratified by **(A)** Nomogram, **(B)** PIAI, **(C)** IPI, and **(D)** PIT risk groups.

According to nomogram, three risk (low-, medium-, high-) categories could be distinguished, and Kaplan-Meier survival curves indicated significant differences in OS incidence among the groups (Log-rank P < 0.001, **Figure 6A**). The categorized distribution included 34.6% in the high-risk group, 33.7% in the medium-risk group, and 33.7% in the low-risk group. The 1-year survival rates were 41.9% for the high-risk group, 93.1% for the medium-risk group, and 95.7% for the low-risk group. Additionally, Kaplan-Meier survival curves indicated significant differences in OS incidence among the groups according to PIAI scoring methods (Log-rank P= 0.024, **Figure 6B**). However, no significant differences in OS incidence were observed among groups when using IPI and PIT scoring methods (Log-rank P= 0.173 and Log-rank P= 0.336, respectively. **Figures 6C, D**). Besides, preliminary calculations (comparing our new model vs. IPI/PIT/PIAI) show NRI of 0.9, 1.0, 0.9, respectively, demonstrating improved risk stratification.

## Discussion

The prognosis of AITL remains poor and heterogeneous. Early identification of high-risk patients is crucial for considering alternative treatments. This necessitates a reliable risk stratification model based on baseline data. Currently, there is limited and often controversial information on prognostic factors in AITL (3, 4). While existing tools like the IPI, PIT and AITL-specific PIAI score are clinically used, they lack functional imaging biomarkers and show suboptimal stratification in our cohort (C-index: 0.64–0.67 in our cohort).

Due to the rarity of AITL, few independent cohorts have validated the prognostic value of PET parameters and established predictive models specifically for AITL. In this study, we comprehensively evaluated the early OS prediction ability based on PET parameters, combined with factors included in clinical prognostic scores IPI, PIT, and PIAI (age, Ann Arbor stage, performance status, extranodal sites, bone marrow involvement, B symptoms, lactate dehydrogenase, platelet), as well as several popular laboratory and pathological indicators (β2MG, albumin, EBV-encoded RNA, and Ki67). Finally, we developed a PET/CT-enhanced prognostic model integrating SUVmax, β2MG, albumin, and platelet count, which significantly outperformed clinical scores in early OS prediction (C-index: 0.78; Log-rank *P*<0.001 vs. PIAI *P*=0.024). Prior PET studies in AITL were limited by small cohorts (n ≤ 56) and focused solely on univariate/multivariate prognostic associations (Hu et al., n=40; Gong et al., n=56; Wang et al., n=23; Seung et al., n=45) (9, 15, 16, 21), our study establishes the first multivariate prognostic model integrating PET metrics with clinical variables.

These results support the satisfactory prognostic value of SUVmax. Interestingly, in this study, lower SUVmax indicated poor early survival, which is inconsistent with Wang’s study of 23 AITL patients, which showed that SUVmax of exodal lesions was an independent predictor of OS in multivariate survival analysis, and high SUVmax (>=4.1) indicates that the OS is poor (15). However, Tsukamoto et al. have shown similar results to ours, indicating that in recurrent intolerant lymphoma, a long axis diameter ≤ 2.5 cm and SUVmax ≤ 6.5 affect PFS (HR: 0.130, *P* = 0.0021 and HR:0.283, *P* = 0.0311) (22). Of course, the results of this study still need to be verified with external data in the future. The superior predictive performance of SUVmax-based assessment compared to that of MTV or TLG is an interesting finding, given that the latter were thought to accurately represent tumor burden (21). One limitation of using MTV and TLG is the lack of standardized methodology, which may affect reproducibility (23). Therefore, further investigation is needed to clarify the precise relative prognostic values of PET parameters.

Albumin levels were important for assessing the general condition of the patient and may reflect exhaustion due to constitutional symptoms. The prognostic significance of low serum albumin has been reported in patients with various lymphoma subtypes, including high-grade lymphoma (24), follicular lymphoma (25), intestinal lymphomas (26), adult T-cell leukemia/lymphoma (27), peripheral T-cell lymphoma (28), and Hodgkin lymphoma (29). In this study, low serum albumin was identified as a poor prognostic factor for early OS.

In terms of β2MG, it has been reported to be adverse prognostic index in various subtypes of lymphoma (30, 31). Several decades ago, Swan F, Jr (32) questioned the Ann Arbor staging system and suggested that serum β2MG levels might offer a more precise system for defining risk groups in large-cell lymphomas. Khouri IF and Rodriguez J (33) also found that high β2MG levels at transplantation were an adverse prognostic factor in patients with diffuse mantle cell lymphoma and peripheral T-cell lymphoma after autologous stem cell transplantation. As a potential prognostic risk factor for AITL, β2MG may operate through several mechanisms: it participates in immune recognition, and loss of functional β2MG through genomic alterations or other mechanisms leads to lack of human leukocyte antigen class I expression and escape from cytotoxic T lymphocyte (34). Additionally, β2MG reflects tumor burden in many tumors (32). Overall, the use of β2MG in our model is reasonable, and it was identified as an independent poor prognostic factor for early overall survival.

This study had the following limitations. Firstly, retrospective design and small cohort size inherently limits generalizability. Although our cohort is larger than that of previous PET/AITL studies, it is still moderate for multivariate modeling. Larger cohorts and prospective multicenter trials are needed in the future. Secondly, despite 1,000-iteration bootstrapping, the modest cohort size necessitates external validation. Thirdly, in this study, only baseline parameters were discussed, and the effect of changes in treatment mode on the prognosis of patients with AILT was not discussed. This prognostic prediction score should be validated in future large-sample, prospective studies to provide valuable information for optimizing treatment decisions and benefiting more AITL patients.

## Conclusions

In conclusion, AITL exhibits a low incidence and poor prognosis, with no specific prognostic model based on PET parameters established to date. In this study, we identified baseline SUVmax, β2MG, albumin, and platelet as independent predictors of early OS in AITL. Hence the new prognostic model may assist in clinical decision-making for AITL patients in clinical practice and also provide a basis for future research. External data validation would be needed to verify the effectiveness of the new scoring system in the future.

## Data Availability

The raw data supporting the conclusions of this article will be made available by the authors, without undue reservation.
