# Supplementary material for: PET/CT-Based prognostic model enhances early survival prediction in angioimmunoblastic t-cell lymphoma
Source: Front Immunol. 2025 Jul 16;16:1607177. doi: 10.3389/fimmu.2025.1607177 (PMC12307193; doi:10.3389/fimmu.2025.1607177)
Supplement: Supplementary file 1 [file DataSheet1.docx]

Supplemental Table 1: Results of univariate and multivariate cox proportional risk models without PET parameters.

| Variables | univariate | | | | |  | multivariate | | | | |
| --- | --- | --- | --- | --- | --- | --- | --- | --- | --- | --- | --- |
|  | β | S.E | Z | *P* | HR (95%CI) |  | β | S.E | Z | *P* | HR (95%CI) |
| EBV-encoded RNA, n(%) |  |  |  |  |  |  |  |  |  |  |  |
| Negative (-) |  |  |  |  | 1.00 (Reference) |  |  |  |  |  |  |
| Positive (+) | 0.59 | 0.36 | 1.64 | 0.101 | 1.81 (0.89 ~ 3.69) |  |  |  |  |  |  |
| Extranodal sites, n(%) |  |  |  |  |  |  |  |  |  |  |  |
| No |  |  |  |  | 1.00 (Reference) |  |  |  |  |  |  |
| Yes | 0.36 | 0.29 | 1.23 | 0.218 | 1.43 (0.81 ~ 2.52) |  |  |  |  |  |  |
| Marrow involvement |  |  |  |  |  |  |  |  |  |  |  |
| No |  |  |  |  | 1.00 (Reference) |  |  |  |  |  |  |
| Yes | 0.11 | 0.37 | 0.30 | 0.763 | 1.12 (0.54 ~ 2.31) |  |  |  |  |  |  |
| Age, n(%) |  |  |  |  |  |  |  |  |  |  |  |
| <=60y |  |  |  |  | 1.00 (Reference) |  |  |  |  |  |  |
| > 60y | 0.54 | 0.31 | 1.73 | 0.083 | 1.71 (0.93 ~ 3.15) |  |  |  |  |  |  |
| Performance status, n(%) |  |  |  |  |  |  |  |  |  |  |  |
| <2 |  |  |  |  | 1.00 (Reference) |  |  |  |  |  |  |
| >=2 | 0.45 | 0.41 | 1.09 | 0.275 | 1.57 (0.70 ~ 3.55) |  |  |  |  |  |  |
| Stage |  |  |  |  |  |  |  |  |  |  |  |
| Ⅰ-Ⅱ |  |  |  |  | 1.00 (Reference) |  |  |  |  |  |  |
| Ⅲ-Ⅳ | 1.42 | 1.01 | 1.41 | 0.159 | 4.15 (0.57 ~ 30.10) |  |  |  |  |  |  |
| Albumin, n(%) |  |  |  |  |  |  |  |  |  |  |  |
| Low (<35.0g/L) | 1.00 | 0.37 | 2.72 | **0.007** | 2.72 (1.32 ~ 5.59) |  | 0.89 | 0.39 | 2.31 | **0.021** | 2.44 (1.15 ~ 5.19) |
| High (>=35.0g/L) |  |  |  |  | 1.00 (Reference) |  |  |  |  |  | 1.00 (Reference) |
| Ki67, n(%) |  |  |  |  |  |  |  |  |  |  |  |
| Low (<0.33) |  |  |  |  | 1.00 (Reference) |  |  |  |  |  |  |
| High (>=0.33) | 0.21 | 0.36 | 0.60 | 0.550 | 1.24 (0.61 ~ 2.50) |  |  |  |  |  |  |
| Lactate dehydrogenase, n(%) |  |  |  |  |  |  |  |  |  |  |  |
| Low (<312.5IU/L) |  |  |  |  | 1.00 (Reference) |  |  |  |  |  |  |
| High (>=312.5IU/L) | 0.44 | 0.31 | 1.45 | 0.148 | 1.56 (0.85 ~ 2.83) |  |  |  |  |  |  |
| β2MG, n(%) |  |  |  |  |  |  |  |  |  |  |  |
| Low (<3.23mg/L) |  |  |  |  | 1.00 (Reference) |  |  |  |  |  | 1.00 (Reference) |
| High (>=3.23mg/L) | 1.04 | 0.36 | 2.93 | **0.003** | 2.83 (1.41 ~ 5.67) |  | 0.99 | 0.36 | 2.79 | **0.005** | 2.70 (1.34 ~ 5.43) |
| B symptom, n(%) |  |  |  |  |  |  |  |  |  |  |  |
| No |  |  |  |  | 1.00 (Reference) |  |  |  |  |  |  |
| Yes | -0.09 | 0.30 | -0.31 | 0.760 | 0.91 (0.51 ~ 1.64) |  |  |  |  |  |  |
| Platelet, n(%) |  |  |  |  |  |  |  |  |  |  |  |
| Low (<150*10^9/L) | 0.96 | 0.32 | 2.99 | **0.003** | 2.61 (1.39 ~ 4.89) |  |  |  |  |  |  |
| High (>=150*10^9/L) |  |  |  |  | 1.00 (Reference) |  |  |  |  |  |  |
| HR: Hazards Ratio, CI: Confidence Interval | | | | | | | | | | | |

Supplemental Table 2: Results of univariate and multivariate cox proportional risk models in training set(n=87).

| Variables | univariate | | | | |  | multivariate | | | | |
| --- | --- | --- | --- | --- | --- | --- | --- | --- | --- | --- | --- |
|  | β | S.E | Z | *P* | HR (95%CI) |  | β | S.E | Z | *P* | HR (95%CI) |
| Age, n(%) |  |  |  |  |  |  |  |  |  |  |  |
| <=60y |  |  |  |  | 1.00 (Reference) |  |  |  |  |  |  |
| > 60y | 0.54 | 0.31 | 1.73 | 0.083 | 1.71 (0.93 ~ 3.15) |  |  |  |  |  |  |
| Performance status, n(%) |  |  |  |  |  |  |  |  |  |  |  |
| <2 |  |  |  |  | 1.00 (Reference) |  |  |  |  |  |  |
| >=2 | 0.45 | 0.41 | 1.09 | 0.275 | 1.57 (0.70 ~ 3.55) |  |  |  |  |  |  |
| Extranodal sites, n(%) |  |  |  |  |  |  |  |  |  |  |  |
| No |  |  |  |  | 1.00 (Reference) |  |  |  |  |  |  |
| Yes | 0.36 | 0.29 | 1.23 | 0.218 | 1.43 (0.81 ~ 2.52) |  |  |  |  |  |  |
| Marrow involvement |  |  |  |  |  |  |  |  |  |  |  |
| No |  |  |  |  | 1.00 (Reference) |  |  |  |  |  |  |
| Yes | 0.11 | 0.37 | 0.30 | 0.763 | 1.12 (0.54 ~ 2.31) |  |  |  |  |  |  |
| EBV-encoded RNA, n(%) |  |  |  |  |  |  |  |  |  |  |  |
| Negative (-) |  |  |  |  | 1.00 (Reference) |  |  |  |  |  |  |
| Positive (+) | 0.59 | 0.36 | 1.64 | 0.101 | 1.81 (0.89 ~ 3.69) |  |  |  |  |  |  |
| Stage |  |  |  |  |  |  |  |  |  |  |  |
| Ⅰ-Ⅱ |  |  |  |  | 1.00 (Reference) |  |  |  |  |  |  |
| Ⅲ-Ⅳ | 1.42 | 1.01 | 1.41 | 0.159 | 4.15 (0.57 ~ 30.10) |  |  |  |  |  |  |
| B symptom, n(%) |  |  |  |  |  |  |  |  |  |  |  |
| No |  |  |  |  | 1.00 (Reference) |  |  |  |  |  |  |
| Yes | -0.09 | 0.30 | -0.31 | 0.760 | 0.91 (0.51 ~ 1.64) |  |  |  |  |  |  |
| Ki67, n(%) |  |  |  |  |  |  |  |  |  |  |  |
| Low (<0.33) |  |  |  |  | 1.00 (Reference) |  |  |  |  |  |  |
| High (>=0.33) | 0.21 | 0.36 | 0.60 | 0.550 | 1.24 (0.61 ~ 2.50) |  |  |  |  |  |  |
| Lactate dehydrogenase, n(%) |  |  |  |  |  |  |  |  |  |  |  |
| Low (<312.5IU/L) |  |  |  |  | 1.00 (Reference) |  |  |  |  |  |  |
| High (>=312.5IU/L) | 0.44 | 0.31 | 1.45 | 0.148 | 1.56 (0.85 ~ 2.83) |  |  |  |  |  |  |
| β2MG, n(%) |  |  |  |  |  |  |  |  |  |  |  |
| Low (<3.23mg/L) |  |  |  |  | 1.00 (Reference) |  |  |  |  |  | 1.00 (Reference) |
| High (>=3.23mg/L) | 1.04 | 0.36 | 2.93 | **0.003** | 2.83 (1.41 ~ 5.67) |  | 0.96 | 0.38 | 2.55 | **0.011** | 2.60 (1.25 ~ 5.43) |
| Platelet, n(%) |  |  |  |  |  |  |  |  |  |  |  |
| Low (<150*10^9/L) | 0.96 | 0.32 | 2.99 | **0.003** | 2.61 (1.39 ~ 4.89) |  | 1.23 | 0.42 | 2.94 | **0.003** | 3.41 (1.50 ~ 7.72) |
| High (>=150*10^9/L) |  |  |  |  | 1.00 (Reference) |  |  |  |  |  | 1.00 (Reference) |
| Albumin, n(%) |  |  |  |  |  |  |  |  |  |  |  |
| Low (<35.0g/L) | 1.00 | 0.37 | 2.72 | **0.007** | 2.72 (1.32 ~ 5.59) |  | 0.95 | 0.42 | 2.27 | **0.023** | 2.58 (1.14 ~ 5.84) |
| High (>=35.0g/L) |  |  |  |  | 1.00 (Reference) |  |  |  |  |  | 1.00 (Reference) |
| SUVMax | -0.05 | 0.02 | -2.09 | **0.037** | 0.95 (0.90 ~ 0.99) |  | -0.12 | 0.03 | -3.80 | **<.001** | 0.88 (0.83 ~ 0.94) |
| MTV | 0.00 | 0.00 | 0.23 | 0.822 | 1.00 (1.00 ~ 1.00) |  |  |  |  |  |  |
| TLG | -0.00 | 0.00 | -1.27 | 0.204 | 1.00 (1.00 ~ 1.00) |  |  |  |  |  |  |
| HR: Hazards Ratio, CI: Confidence Interval | | | | | | | | | | | |


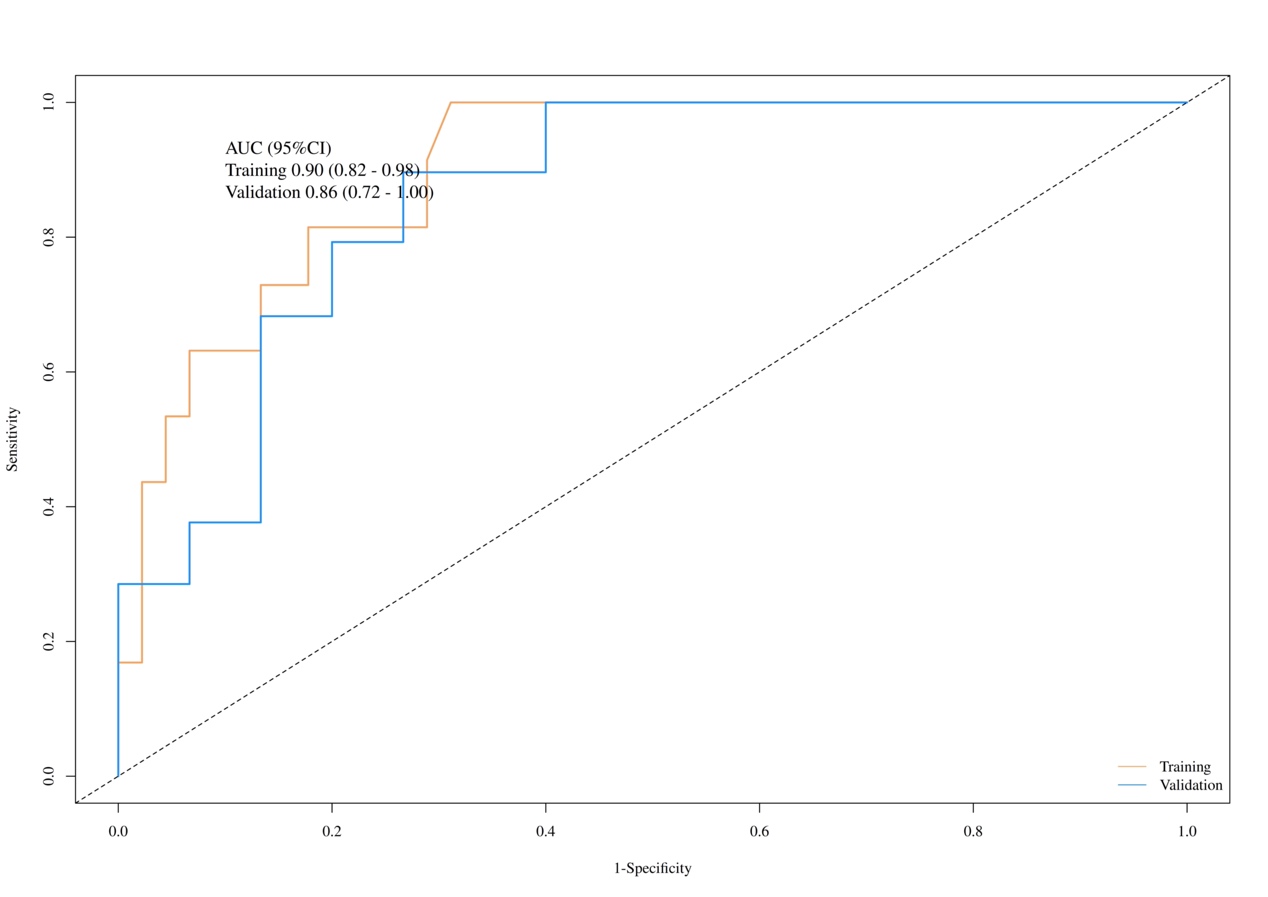


Supplemental Figure1: ROC curve and AUC for predicting the early OS of AITL patients in training and validation sets.

Supplemental Table 3: Results of univariate and multivariate cox proportional risk models with interim PET/CT parameters.

| Variables | univariate | | | | |  | multivariate | | | | | |
| --- | --- | --- | --- | --- | --- | --- | --- | --- | --- | --- | --- | --- |
|  | β | S.E | Z | *P* | HR (95%CI) |  | β | S.E | Z | *P* | HR (95%CI) | |
| Performance status, n(%) |  |  |  |  |  |  |  |  |  |  |  | |
| <2 |  |  |  |  | 1.00 (Reference) |  |  |  |  |  |  | |
| >=2 | 0.45 | 0.41 | 1.09 | 0.275 | 1.57 (0.70 ~ 3.55) |  |  |  |  |  |  | |
| Stage |  |  |  |  |  |  |  |  |  |  |  | |
| Ⅰ-Ⅱ |  |  |  |  | 1.00 (Reference) |  |  |  |  |  |  | |
| Ⅲ-Ⅳ | 1.42 | 1.01 | 1.41 | 0.159 | 4.15 (0.57 ~ 30.10) |  |  |  |  |  |  | |
| Extranodal sites, n(%) |  |  |  |  |  |  |  |  |  |  |  | |
| No |  |  |  |  | 1.00 (Reference) |  |  |  |  |  |  | |
| Yes | 0.36 | 0.29 | 1.23 | 0.218 | 1.43 (0.81 ~ 2.52) |  |  |  |  |  |  | |
| Marrow involvement |  |  |  |  |  |  |  |  |  |  |  | |
| No |  |  |  |  | 1.00 (Reference) |  |  |  |  |  |  | |
| Yes | 0.11 | 0.37 | 0.30 | 0.763 | 1.12 (0.54 ~ 2.31) |  |  |  |  |  |  | |
| Age,  n(%) |  |  |  |  |  |  |  |  |  |  |  | |
| <=60y |  |  |  |  | 1.00 (Reference) |  |  |  |  |  |  | |
| > 60y | 0.54 | 0.31 | 1.73 | 0.083 | 1.71 (0.93 ~ 3.15) |  |  |  |  |  |  | |
| EBV-encoded RNA, n(%) |  |  |  |  |  |  |  |  |  |  |  | |
| Negative (-) |  |  |  |  | 1.00 (Reference) |  |  |  |  |  |  | |
| Positive (+) | 0.59 | 0.36 | 1.64 | 0.101 | 1.81 (0.89 ~ 3.69) |  |  |  |  |  |  | |
| β2MG, n(%) |  |  |  |  |  |  |  |  |  |  |  | |
| Low (<3.23mg/L) |  |  |  |  | 1.00 (Reference) |  |  |  |  |  | 1.00 (Reference) | |
| High (>=3.23mg/L) | 1.04 | 0.36 | 2.93 | **0.003** | 2.83 (1.41 ~ 5.67) |  | 0.96 | 0.38 | 2.55 | **0.011** | 2.60 (1.25 ~ 5.43) | |
| Lactate dehydrogenase, n(%) |  |  |  |  |  |  |  |  |  |  |  | |
| Low (<312.5IU/L) |  |  |  |  | 1.00 (Reference) |  |  |  |  |  |  | |
| High (>=312.5IU/L) | 0.44 | 0.31 | 1.45 | 0.148 | 1.56 (0.85 ~ 2.83) |  |  |  |  |  |  | |
| Platelet, n(%) |  |  |  |  |  |  |  |  |  |  |  | |
| Low (<150*10^9/L) | -0.96 | 0.32 | -2.99 | **0.003** | 0.38 (0.20 ~ 0.72) |  |  | 0.42 | -2.94 | **0.003** | 0.29 (0.13 ~ 0.66) | |
| High (>=150*10^9/L) |  |  |  |  | 1.00 (Reference) |  |  |  |  |  | 1.00 (Reference) | |
| Albumin, n(%) |  |  |  |  |  |  |  |  |  |  |  | |
| Low (<35.0g/L) | 1.00 | 0.37 | 2.72 | **0.007** | 2.72 (1.32 ~ 5.59) |  | 0.95 | 0.42 | 2.27 | **0.023** | 2.58 (1.14 ~ 5.84) | |
| High (>=35.0g/L) |  |  |  |  | 1.00 (Reference) |  |  |  |  |  | 1.00 (Reference) | |
| Ki67, n(%) |  |  |  |  |  |  |  |  |  |  |  | |
| Low (<0.33) |  |  |  |  | 1.00 (Reference) |  |  |  |  |  |  | |
| High (>=0.33) | 0.21 | 0.36 | 0.60 | 0.550 | 1.24 (0.61 ~ 2.50) |  |  |  |  |  |  | |
| B symptom, n(%) |  |  |  |  |  |  |  |  |  |  |  | |
| No |  |  |  |  | 1.00 (Reference) |  |  |  |  |  |  | |
| Yes | -0.09 | 0.30 | -0.31 | 0.760 | 0.91 (0.51 ~ 1.64) |  |  |  |  |  |  | |
| SUVmax | -0.05 | 0.02 | -2.09 | **0.037** | 0.95 (0.90 ~ 0.99) |  | -0.12 | 0.03 | -3.80 | **<.001** | 0.88 (0.83 ~ 0.94) | |
| MTV | 0.00 | 0.00 | 0.23 | 0.822 | 1.00 (1.00 ~ 1.00) |  |  |  |  |  |  | |
| TLG | -0.00 | 0.00 | -1.27 | 0.204 | 1.00 (1.00 ~ 1.00) |  |  |  |  |  |  | |
| ΔSUVmax | -0.05 | 0.03 | -1.67 | 0.095 | 0.95 (0.90 ~ 1.01) |  |  |  |  |  |  | |
| ΔMTV | -0.00 | 0.00 | -0.74 | 0.459 | 1.00 (1.00 ~ 1.00) |  |  |  |  |  |  | |
| ΔTLG | -0.00 | 0.00 | -1.44 | 0.149 | 1.00 (1.00 ~ 1.00) |  |  |  |  |  |  | |
| HR: Hazards Ratio, CI: Confidence Interval | | | | | | | | | | | |  |


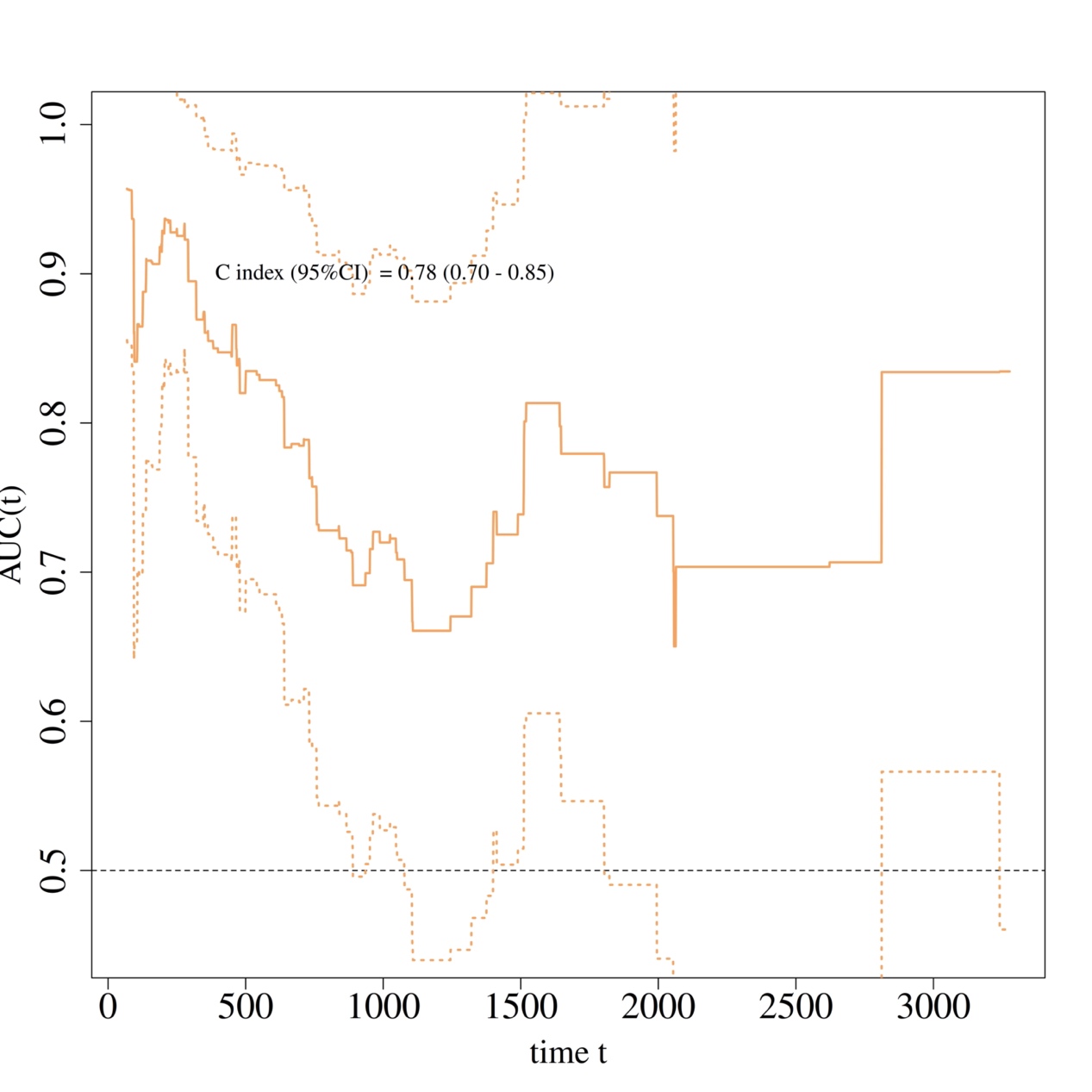


Supplemental Figure2: Time-dependent ROC curve for predicting the early OS of AITL patients in total set.

Supplemental Table 4: Results of univariate and multivariate cox proportional risk models with interim PET/CT parameters and T-cell subset data.

| Variables | univariate | | | | |  | multivariate | | | | |
| --- | --- | --- | --- | --- | --- | --- | --- | --- | --- | --- | --- |
|  | β | S.E | Z | *P* | HR (95%CI) |  | β | S.E | Z | *P* | HR (95%CI) |
| Age,  n(%) |  |  |  |  |  |  |  |  |  |  |  |
| <=60y |  |  |  |  | 1.00 (Reference) |  |  |  |  |  |  |
| > 60y | 0.60 | 0.32 | 1.88 | 0.060 | 1.82 (0.98 ~ 3.38) |  |  |  |  |  |  |
| Performance status, n(%) |  |  |  |  |  |  |  |  |  |  |  |
| <2 |  |  |  |  | 1.00 (Reference) |  |  |  |  |  |  |
| >=2 | 0.45 | 0.41 | 1.09 | 0.275 | 1.57 (0.70 ~ 3.55) |  |  |  |  |  |  |
| Stage |  |  |  |  |  |  |  |  |  |  |  |
| Ⅰ-Ⅱ |  |  |  |  | 1.00 (Reference) |  |  |  |  |  |  |
| Ⅲ-Ⅳ | 1.42 | 1.01 | 1.41 | 0.159 | 4.15 (0.57 ~ 30.10) |  |  |  |  |  |  |
| Extranodal sites, n(%) |  |  |  |  |  |  |  |  |  |  |  |
| No |  |  |  |  | 1.00 (Reference) |  |  |  |  |  |  |
| Yes | 0.36 | 0.29 | 1.23 | 0.218 | 1.43 (0.81 ~ 2.52) |  |  |  |  |  |  |
| Marrow involvement |  |  |  |  |  |  |  |  |  |  |  |
| No |  |  |  |  | 1.00 (Reference) |  |  |  |  |  |  |
| Yes | 0.11 | 0.37 | 0.30 | 0.763 | 1.12 (0.54 ~ 2.31) |  |  |  |  |  |  |
| EBV-encoded RNA, n(%) |  |  |  |  |  |  |  |  |  |  |  |
| Negative (-) |  |  |  |  | 1.00 (Reference) |  |  |  |  |  |  |
| Positive (+) | 0.59 | 0.36 | 1.64 | 0.101 | 1.81 (0.89 ~ 3.69) |  |  |  |  |  |  |
| Albumin, n(%) |  |  |  |  |  |  |  |  |  |  |  |
| Low (<35.0g/L) | 1.00 | 0.37 | 2.72 | **0.007** | 2.72 (1.32 ~ 5.59) |  |  |  |  |  |  |
| High (>=35.0g/L) |  |  |  |  | 1.00 (Reference) |  |  |  |  |  |  |
| B symptom, n(%) |  |  |  |  |  |  |  |  |  |  |  |
| No |  |  |  |  | 1.00 (Reference) |  |  |  |  |  |  |
| Yes | -0.09 | 0.30 | -0.31 | 0.760 | 0.91 (0.51 ~ 1.64) |  |  |  |  |  |  |
| Ki67, n(%) |  |  |  |  |  |  |  |  |  |  |  |
| Low (<0.33) |  |  |  |  | 1.00 (Reference) |  |  |  |  |  |  |
| High (>=0.33) | 0.21 | 0.36 | 0.60 | 0.550 | 1.24 (0.61 ~ 2.50) |  |  |  |  |  |  |
| β2MG, n(%) |  |  |  |  |  |  |  |  |  |  |  |
| Low (<3.23mg/L) |  |  |  |  | 1.00 (Reference) |  |  |  |  |  |  |
| High (>=3.23mg/L) | 1.04 | 0.36 | 2.93 | **0.003** | 2.83 (1.41 ~ 5.67) |  |  |  |  |  |  |
| Lactate dehydrogenase, n(%) |  |  |  |  |  |  |  |  |  |  |  |
| Low (<312.5IU/L) |  |  |  |  | 1.00 (Reference) |  |  |  |  |  |  |
| High (>=312.5IU/L) | 0.44 | 0.31 | 1.45 | 0.148 | 1.56 (0.85 ~ 2.83) |  |  |  |  |  |  |
| Platelet, n(%) |  |  |  |  |  |  |  |  |  |  |  |
| Low (<150*10^9/L) | -0.96 | 0.32 | -2.99 | **0.003** | 0.38 (0.20 ~ 0.72) |  | -2.13 | 0.52 | -4.12 | **<.001** | 0.12 (0.04 ~ 0.33) |
| High (>=150*10^9/L) |  |  |  |  | 1.00 (Reference) |  |  |  |  |  | 1.00 (Reference) |
| CD3+ | -0.03 | 0.01 | -1.98 | **0.047** | 0.97 (0.95 ~ 0.99) |  |  |  |  |  |  |
| CD3+/CD4+ | -0.00 | 0.02 | -0.25 | 0.806 | 1.00 (0.97 ~ 1.03) |  |  |  |  |  |  |
| CD3+/CD8+ | -0.03 | 0.02 | -1.84 | 0.065 | 0.97 (0.94 ~ 1.00) |  |  |  |  |  |  |
| CD4+/CD25+ | 0.10 | 0.04 | 2.19 | **0.028** | 1.10 (1.01 ~ 1.20) |  | 0.12 | 0.04 | 2.75 | **0.006** | 1.13 (1.04 ~ 1.23) |
| CD8+/CD28- | -0.01 | 0.02 | -0.40 | 0.687 | 0.99 (0.96 ~ 1.03) |  |  |  |  |  |  |
| CD8+/CD28+ | -0.06 | 0.04 | -1.61 | 0.108 | 0.94 (0.87 ~ 1.01) |  |  |  |  |  |  |
| SUVmax | -0.05 | 0.02 | -2.09 | **0.037** | 0.95 (0.90 ~ 0.99) |  | -0.13 | 0.04 | -2.99 | **0.003** | 0.88 (0.80 ~ 0.96) |
| MTV | 0.00 | 0.00 | 0.23 | 0.822 | 1.00 (1.00 ~ 1.00) |  |  |  |  |  |  |
| TLG | -0.00 | 0.00 | -1.27 | 0.204 | 1.00 (1.00 ~ 1.00) |  |  |  |  |  |  |
| ΔSUVmax | -0.05 | 0.03 | -1.67 | 0.095 | 0.95 (0.90 ~ 1.01) |  |  |  |  |  |  |
| ΔMTV | -0.00 | 0.00 | -0.74 | 0.459 | 1.00 (1.00 ~ 1.00) |  |  |  |  |  |  |
| ΔTLG | -0.00 | 0.00 | -1.44 | 0.149 | 1.00 (1.00 ~ 1.00) |  |  |  |  |  |  |
| HR: Hazards Ratio, CI: Confidence Interval | | | | | | | | | | | |
